# Is Every Patient Followed up as a Papillary Thyroid Cancer Patient Really That?

**DOI:** 10.4274/Mirt.022866

**Published:** 2012-04-01

**Authors:** Ummuhan Abdulrezzak, Ahmet Tutus, Mustafa Kula, Figen Öztürk, Işın Soyuer

**Affiliations:** 1 Erciyes University School of Medicine, Department of Nuclear Medicine, Kayseri, Turkey; 2 Erciyes University School of Medicine, Department of Pathology, Kayseri, Turkey

**Keywords:** Thyroid nodule, carcinoma, renal cell, Positron-emission tomography

## Abstract

We report the case of a 64-year-old man followed up for two years as suffering from differentiated thyroid cancer (DTC). In the patient’s follow up, despite thyroglobulin level and I-131 whole body scan results being normal, metastases were identified at the 4th thoracic vertebra corpus by MR. Histopathological findings were carcinoma metastases. F-18 FDG PET/CT showed increased metabolic activity in the right renal mass, bilaterally in the surrenal gland, multiple lymph nodes in the thoracic and abdominal para-aortic region and in multiple vertebral and pelvic bones. An excisional biopsy of the right renal mass was reported as renal cell carcinoma. Immunohistochemical staining performed retrospectively to the first thyroid preparation showed renal cell carcinoma metastases. Consequently, any patient who presents with a thyroid nodule can also be considered as possibly suffering from metastatic disease. F-18 FDG PET/CT can provide valuable information in finding the primary focus and metastases.

**Conflict of interest:**None declared.

## INTRODUCTION

Although papillary and follicular thyroid cancers make up the majority of cases of thyroid malignancy, other types of malignancies and metastases are rather rare (1). The therapy strategies of papillary and follicular cancers called as “differentiated thyroid cancers (DTC)” and other types of thyroid cancers are different. DTC is managed initially by surgical intervention (total or near total thyroidectomy and if indicated, lymphadenectomy) followed by permanent thyroid ablation with radioiodine therapy (I-131) (2,3,4). In other thyroid cancers, treatment varies according to cancer type. Some cases reported in the literature that are metastatic to the thyroid may masquerade as primary thyroid neoplasms (5,6). Therefore, diagnosis of these cancers needs to be based on a detailed clinical examination and combination of pathological and imaging modalities

## CASE REPORT

A 64-year-old man presenting with a palpable mass in the right lobe of the thyroid gland demonstrated a hypoechoic nodule and bilateral cervical lymphadenopathy in ultrasonography. Fine-needle aspiration biopsy revealed the doubtful-malign features of a thyroid follicular lesion. 

A near total thyroidectomy and bilateral 2-7th level lymph node dissection were performed and the final histological examination disclosed a multicentricitic papillary thyroid carcinoma (1.7 cm in diameter in the right lobe and occult carcinoma focus in the left lobe) and 12/55 number of metastatic lymph nodes. At that time, immunohistochemical staining was not applied to the specimens. Two months later, I-131 ablation therapy (5.55 GBq) was performed after the withdrawal of TSH-suppressive L-thyroxine therapy for four weeks (TSH: 69 μIU/mL). A post-treatment I-131 whole body scan (WBS) demonstrated some activity in the remnants and no distant metastases. After radioiodine remnant ablation an I-131 WBS was performed at the 6^th^ and 20^th^ months to evaluate the success of treatment and did not demonstrate any pathological findings. 

After two years, the patient applied to the outpatient clinic with complaints of back pain. On finding a solid lesion at the 4th thoracic vertebra in MR, an excisional biopsy was performed on the vertebra corpus and the results were reported as carcinoma metastases. The vertebral metastases were thought to be thyroid cancer metastases because the patient had been followed up for papillary thyroid cancer. At that time, his TSH-stimulated (TSH: 62 μIU/mL) thyroglobulin (Tg) was below the detection limit (<0.5 ng/mL), thyroglobulin antibody (Tg Ab) was low (<10 ng/mL) and I-131 WBS were negative. 

**Figure 1:**
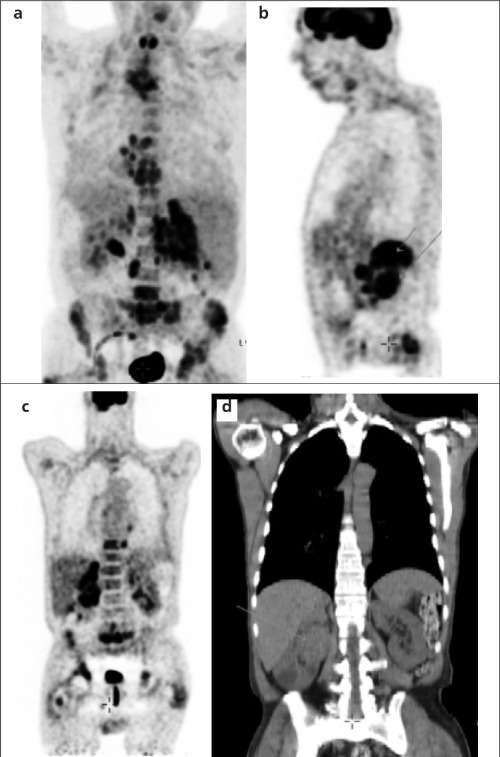
F-18 FDG PET/CT images showing thoracic and abdominalmultiple para-aortic hypermetabolic lymph nodes, increased F-18 FDGuptake in multiple foci of thoracic and lumbar vertebrae, and in thepelvic bones with osteolytic lesions showing up as patches on CT(a: MIP image; b: sagittal section; c: coronal section and d: coronal CTsection). There was clearly a pathologic uptake of the soft tissuelesion originating from the right kidney measuring 6x5x3 cm andbilaterally in the surrenal glands (left thickened surrenal gland measuring2x1x1 cm, and right surrenal gland measuring 12x9x4 cm)

The patient was then examined with F-18 FDG PET/CT for any further metastases. Increased accumulation of F-18 FDG was seen in the thoracic and abdominal multiple paraaortic lymph nodes. PET/CT demonstrated multiple foci of increased F-18 FDG uptake in the thoracic and lumbar vertebrae and in the pelvic bones with osteolytic lesions showing up as patches on CT. In addition, there was clearly a bilateral pathologic uptake in the surrenal glands. However, the most important finding was a soft tissue lesion measuring 6x5x3 cm which originated from the right kidney with intense F-18 FDG uptake (Figure 1). 

**Figure 2:**
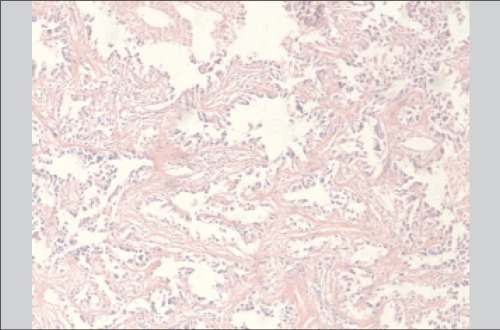
Immunohistochemical staining of the right kidney lesionwith hematoxylin and eosin (H&E) revealed renal cell carcinoma(x40)

An excisional biopsy was then taken from the right kidney lesion. The pathologic diagnosis was reported as renal cell carcinoma (Figure 2). 

**Figure 3:**
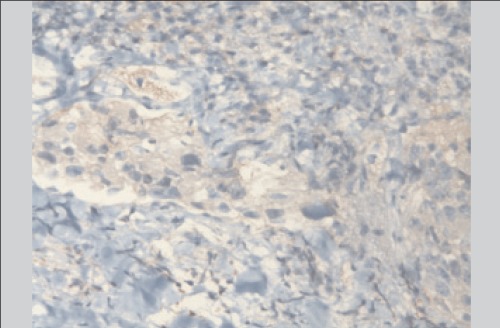
Immunohistochemical staining of the original thyroid tissuespecimens showed that tumor cells had cytoplasmic positivity ina case of “metastatic” renal cell carcinoma for CD10 antibody (x100)

At this point, the original thyroid tissue and vertebral specimens were reanalyzed. Immunohistochemical staining was performed to characterize the lesions. Tumor cells were focal positive for CD10 and EMA and negative for thyroglobulin, TTF1 (thyroid transcription factor 1) and calcitonin, suggestive of metastatic renal cell carcinoma (Figure 3). The same immunostaining results were also obtained from the renal specimen. Consequently, in this patient, the primary diagnosis and therapy strategy were entirely changed as a result of F-18 FDG PET/CT.

## LITERATURE REVIEW AND DISCUSSION

The evaluation of the clinical situation in any patient with suspected thyroid disease should be based on a detailed case history, followed by a careful physical examination. Patients with DTC have a relatively good overall prognosis (7). However, local recurrences and distant metastases are not uncommon, particularly during the first years of follow-up, but sometimes they occur many years later (8,9). Therefore, long-term monitoring of DTC recurrence and metastases is essential throughout the patient’s life. Serum Tg measurement and I-131 WBS are generally used in the follow-up of patients with DTC to detect any local recurrences or metastases (10,11) However, in some unusual situations the I-131 WBS and thyroglobulin level may be normal even when recurrent or metastatic disease exists (12,13). Hence, conventional screening methods are not sufficient in cases of suspected clinical situations occurring in the follow-up of DTC patients, and additional methods are necessary to detect early recurrences and metastases. 

The fusion of metabolic and morphologic information with PET/CT enables greater diagnostic accuracy, reduces pitfalls and changes therapeutic strategies in a reasonable number of cancer patients. Many F-18 FDG PET studies have been performed in patients with differentiated thyroid cancer so far. However, most of these studies have been performed in cases with elevated Tg levels and a negative I-131 scan (14,15). Elevated plasma Tg is a specific marker for persistent or recurrent disease after total thyroidectomy and radioiodine ablation. However, the follow-up of thyroid cancer should not rely only upon Tg determination, even after suppression therapy withdrawal. The presence of anti-Tg antibodies (Tg Ab) usually invalidates the serum Tg result because any change in the concentration or affinity of Tg Ab has the potential to alter the measured Tg value.16 In addition, in the study by Brendel et al., it was reported that in 8.5% of DTC cases without anti-Tg antibodies, very low Tg levels were associated with metastases. 17 Thus, in the follow-up of thyroid cancer, Tg and Tg Ab can not always be used as reliable tumor markers. In our case, while the patient was followed up for two years as suffering from DTC, another cancer, renal cell carcinoma, was diagnosed by F-18 FDG PET/CT and was confirmed histopathologically.In the follow-up, owing to negative Tg values and I-131 scans, the patient was thought to be disease free; however, F-18 FDG PET affected the primary diagnosis and management of this patient. Based on data in the literature, F-18 FDG PET/CT does not have routine clinical indication in the follow-up of DTC patients. As an excellent diagnostic tool, F-18 FDG PET/CT is justified only in patients with a negative I-131 scan when Tg is elevated. However, when Tg is not detectable there may still be recurrences, metastases or another clinical situation. 

Metastases to the thyroid are uncommon among the reported clinical cases, but are more frequent in autopsy series.18 The thyroid involvement rate by non-thyroid malignancies has been reported as between 1.9-24% (19). The most common sites of the primary tumors are the kidney, breast and lung and in some cases they are detected only at autopsy (20). Renal cell carcinoma metastatic to the thyroid may be misperceived as a primary thyroid neoplasm (21,22). A history of prior nephrectomy or complaint associated with the kidney and the presence of thyroid nodules may connote metastatic renal cell carcinoma. In our patient, there was no history or complaint in connection with the kidney. The metastatic tumor in the thyroid gland was the initial presentation of renal carcinoma but the patient mistakenly followed up for two years as suffering from papillary thyroid cancer. Immunohistochemistry is also a helpful adjunct in the evaluation of thyroid nodules to differentiate primary thyroid malignancy from metastatic disease. It has been shown that tumor cells that are negative for thyroglobulin, TTF1 and calcitonin to rule out primary thyroid carcinoma (23). These findings suggest metastatic thyroid disease and at this step, F-18 FDG PET/CT must be incorporated into the cycle to find the primary malignancy and metastases that can exist in any region of the body.

We concluded that, in any patient who presents with a thyroid nodule, possible metastatic disease from a previous primary tumor should be considered and a full clinical history must be obtained. If the findings are suspicious, immunohistochemical staining can be helpful. On the other hand, it should be emphasized that F-18 FDG PET/CT can not only be used for thyroid disease patients with an elevated Tg and I-131 negative scan, but it can also be used for any suspected evidence in the initial diagnosis and follow-up of the patients.
